# Q-LBR: Q-Learning Based Load Balancing Routing for UAV-Assisted VANET

**DOI:** 10.3390/s20195685

**Published:** 2020-10-05

**Authors:** Bong-Soo Roh, Myoung-Hun Han, Jae-Hyun Ham, Ki-Il Kim

**Affiliations:** 1 Agency for Defense Development, Daejeon 34186, Korea; saintroh@add.re.kr (B.-S.R.); mengddor@add.re.kr (M.-H.H.); mjhham@add.re.kr (J.-H.H.); 2 Department of Computer Science and Engineering, Chungnam National University, Daejeon 34134, Korea

**Keywords:** MANET, VANET, UAV relay, load balancing, routing, Q learning

## Abstract

Although various unmanned aerial vehicle (UAV)-assisted routing protocols have been proposed for vehicular ad hoc networks, few studies have investigated load balancing algorithms to accommodate future traffic growth and deal with complex dynamic network environments simultaneously. In particular, owing to the extended coverage and clear line-of-sight relay link on a UAV relay node (URN), the possibility of a bottleneck link is high. To prevent problems caused by traffic congestion, we propose Q-learning based load balancing routing (Q-LBR) through a combination of three key techniques, namely, a low-overhead technique for estimating the network load through the queue status obtained from each ground vehicular node by the URN, a load balancing scheme based on Q-learning and a reward control function for rapid convergence of Q-learning. Through diverse simulations, we demonstrate that Q-LBR improves the packet delivery ratio, network utilization and latency by more than 8, 28 and 30%, respectively, compared to the existing protocol.

## 1. Introduction

The vehicular ad hoc network (VANET), a special type of mobile ad hoc network (MANET), has been investigated to provide the infrastructure of a new service paradigm through self-organizing networks that exist between vehicles. However, it still experiences difficulty in routing with easily disconnected features that are associated with dynamic wireless environments in mobile network topologies. To overcome this problem, the deployment of unmanned aerial vehicles (UAVs) via the cooperation of vehicles has been considered.

Several methods have recently been developed in the literature for UAV-assisted network protocols that address the issues of high mobility in the network and unpredictable change in topology of the mobile nodes [1,2,3,4,5,6]. Unlike a fixed ground relay station, a UAV relay node (URN) moves along with the ground vehicular nodes (GVNs) to support a reliable network through a continuous line-of-sight (LoS) link. In addition, considering the characteristics through which MANET is temporarily constructed and operated, this is an extremely economical solution compared to the construction of a ground infrastructure. In the case of a VANET, in particular, the relay node is faced with the risk of a broken link that can be caused by mobility, and nonline-of-sight (NLoS) can occur more frequently than in a general MANET. Therefore, a UAV-assisted relay can be a more useful tool when operating in a VANET environment. Because the UAV relay path is most likely to be the best approach in terms of link quality and the number of hops, it is highly likely that a bottleneck of the URN will occur from the existing routing protocol when the network is congested. This bottleneck can degrade the transmission efficiency of the UAV and, in the case of Carrier Sense Multiple Access/Collision Avoidance (CSMA/CA MAC, Ex. 802.11p), the channel access opportunities of all the ground nodes can be lost. In addition, the spatial frequency reusability of the ground node can be lowered, resulting in a decrease in the overall network performance. However, if a UAV relay node (URN) is operated only as a backup path when a ground link disconnection occurs, the URN resources are wasted. Therefore, to increase the efficiency of a URN, it is necessary to design a structure that can handle the maximum acceptable traffic while maintaining a certain level of ground network load.

In the design of the Q-learning based load balancing routing (Q-LBR), a URN uses an overhearing technique based on the broadcast nature of wireless media to recognize the ground network load in the message transmitted between the GVNs. The URN then distributes the UAV routing policy area (URPA) information based on the Q-learning method through broadcast messages such as Hello. The GVNs decide whether to use the air relay path through the received URPA and the current UAV relay load, and the URN continuously maximizes the network throughput even when the wireless environment is dynamically changing. We propose a Q-LBR system for load balancing in a UAV-assisted VANET. This provides a method for URN to handle the maximum traffic acceptable while maintaining a certain level of ground network load. 

The main contributions of this study are fourfold:

(1) We propose a low-overhead technique for estimating the network load through the queue status obtained by the URN from each GVN. To estimate a network load with low overhead, we design a technique using overhearing and broadcast messages received from the GVNs. This is possible because the URN can cover an area wider than that of the GVNs.
(2) We propose a load balancing scheme based on Q-learning, which can enable dynamic network load control within the usable capacity of the URN. Q-LBR defines the URPA when considering the traffic characteristics and the existence of a route independently from the ground network routing.
(3) We propose a reward control function (RCF) to enable rapid learning feedback of the reward values in consideration of a dynamic network environment. Q-LBR adjusts the reward value based on the URN load and ground network congestion.
(4) We implemented the Q-LBR on a network-level simulator using the Riverbed Modeler (formerly OPNET) and experimentally evaluated its performance. Our evaluation results showed that Q-LBR achieved a significantly better packet delivery ratio (PDR) and network utilization and latency according to the traffic load conditions than existing algorithms and Q-LBR without Q-learning.

## 2. Related Studies

This section describes the UAV-aided routing and load balancing routing as well as the Q-learning-based routing, which have been designed to enhance the existing VANETs. Their respective limitations are then addressed to clarify our motivation for the Q-LBR design.

### 2.1. UAV-Assisted Routing Protocols

Research on various applications using drones has been rapidly increasing, and that on UAV-assisted VANETs is also actively underway. Load carry and deliver routing (LCAD) [3] was proposed for static single-hop routing for UAVs to assist ground nodes. LCAD provides a load-carry-delivery mechanism for enhancing connectivity during the data delivery process of a sparsely connected network by applying the disruption tolerant network (DTN) concept. As a drawback, LCAD does not consider the traffic characteristics of a URN bottleneck. UAV-assisted VANET routing (UVAR) [4] and its extension (U2RV, UAV-assisted reactive routing protocol for VANETs) [5] utilize reactive multipath routing for UAV-assisted routing. UVAR and U2RV are based on four main processes: discovery, selection, data delivery and maintenance. These protocols calculate a multi-criteria score for considering the highest degree of connectivity and the shortest distance, as well as the minimum delay to the target destination. In terms of the selection process, the score is calculated for every discovered path by combining several metrics. As a result, the best scored path will be selected, and it is difficult to guarantee the service Quality of service (QoS) when the traffic is concentrated on the corresponding path. Therefore, similar to LCAD, these protocols do not consider the traffic characteristics or URN bottlenecks. A multi-UAV-aided MEC architecture has been proposed [6] as a joint multi-UAV deployment and task scheduling optimization for IoT networks. This paper proposed the task scheduling method using deep reinforcement learning in terms of the role of the Multi-access Edge Computing (MEC) node. However, in terms of the relay node of the URN, routing and load balancing for the traffic priority and characteristics were not considered.

### 2.2. Load-Balancing Routing Protocols

With the rapid increase in the use of VANETs, and increased demand of networked vehicles for a wide range of services and better information, load balancing has become an essential and important research area. Efficient load balancing ensures efficient resource utilization and enhances the overall performance of the network system. UAV-aided cross-layer routing (UCLR) [7] is a cross-layer routing and load-balancing algorithm that considers the UAV relay based on Open Shorstest Path First-MANET Designed Router (OSPF-MDR). The routing metric of UCLR is calculated using the packet error rate (PER), and load balancing is adjusted using a static threshold of the queue length. Although UCLR handles dynamic UAV traffic load issues between the URN and GVNs, the main drawback of the UCLR is its static load control with a dynamic network environment. In a UAV-assisted VANET environment there are several moving GVNs, and the changes in the traffic patterns are also extremely rapid. Therefore, there is a need for a dynamic load control scheme capable of responding to rapidly changing network environments. Moreover, UCLR does not consider a method for improving the utilization of the UAV throughput. A hierarchical routing scheme with load balancing (HRLB) has been proposed [8] as a hierarchical geography routing protocol for software-defined VANETs. HRLB constructs a path cost function with load balancing and maintains two paths with minimal costs from the selected grids. This protocol considers the load only from the GVN and disregards the UAV-assisted relay. A queue utilization routing algorithm (QURA) has been proposed [9] as a machine learning-based routing scheme for QoS routing. This protocol applies an artificial neural network (ANN) to routing and selects the next hop according to a queue utilization prediction (QUP). However, supervised learning has a problem in that it is difficult to create the training data and the label in dynamic network topology. Table 1 summarizes the characteristics of the UAV-assisted routing and load balancing protocols. By reviewing these protocols, we can state that most of the proposed routing protocol techniques designed for UAV-assisted VANET disregarded the traffic characteristics and dynamic load balancing in congested network environments. In addition, these routing protocols do not consider traffic bottlenecks owing to a better link quality and hop count compared to the ground network of a UAV relay node.

To address the aforementioned problems, we propose a new load-balancing routing scheme that is capable of achieving efficient operation of UAV relay nodes in consideration of the traffic characteristics. In addition, we use the Q-learning algorithm, which improves the convergence speed of the reward function for dynamic load control.

### 2.3. Q-Learning-Based Routing Protocols

In recent years, artificial intelligence techniques, which include machine learning, have attracted a significant amount of interest from researchers of various fields [8]. Among such techniques, reinforcement learning (RL) is being investigated in wireless systems because it provides a solution to optimize the system parameters by learning the surrounding area in a dynamic and complicated wireless environment [10,11,12]. Q-learning is a representative RL, and studies on using this approach to allocate routing policies in a dynamically changing network environment have been conducted. The Q-learning algorithm [13] solves this problem by utilizing the following Q-value update equation:

(1) $$Q\left(s_{t+1},\alpha_{t+1}\right)\leftarrow \left(1-\alpha \right)Q\left(s_{t},\alpha_{t}\right)+\alpha \left\{f_{r}\left(s_{t},\alpha_{t}\right)+\gamma max_{\alpha^{\prime }}\left(Q\left(s_{t},\alpha_{t}\right),\alpha^{\prime }\right)\right\},$$

where $Q\left(s_{t},\alpha_{t}\right)$ is the Q-value of the current state $s_{t}$ when action *α* is selected at time *t*, $f_{r}\left(s_{t},\alpha_{t}\right)$ represents the reward function when state $s_{t}$ selects action $\alpha_{t}$, and max($Q\left(s_{t},\alpha_{t}\right)$, *α’*) is the maximum possible Q-value in the next state $s_{t+1}$ when possible action *α’* is selected. The learning rate α and discount factor γ have values between zero and one. As an advantage of Q-learning, it can be used to design optimal policy functions even in unknown environments. In general, a wireless network environment is extremely complex and difficult to predict and, therefore, it is considered that reinforcement learning such as Q-learning is more suitable than supervised learning.

There are several noteworthy studies on Q-learning-based routing protocols. Q-Geo [14] proposed an ad hoc routing method based on geographic information through Q-learning in an unmanned robotic network. This algorithm enables network enhancement using local information without full network knowledge by calculating the packet travel speed. The energy-aware QoS routing protocol (EQR-RL) [15] uses a reinforcement learning algorithm and the reinforcement learning based geographic routing (RLGR) [16] are proposed methods for applying Q-learning in routing decisions for a network lifetime enhancement in a Wireless sensor network (WSN). Q-learning based fuzzy logic [17] for multi-objective routing algorithm is proposed as a method for flying ad hoc networks (FANET).

Although there have been numerous studies applying Q-learning, results for UAV-assisted VANET are yet to be presented. In addition, the key issue for applying RL to a rapidly changing network environment is solving the convergence speed problem. Specifically, RL is based on the results of experiences acquired through exploration and, thus, it sometimes takes significant trial and error to obtain meaningful results. Likewise, until recently, reinforcement learning in the field of networking has not been considered.

## 3. System Model and Assumptions

In this section, we describe the system model and some key network assumptions. Q-LBR assumes that the UAV relay node has a low and constant altitude during flight to be able to relay with vehicles on the ground, and that all network nodes have the same RF performance. However, URN has a relatively low signal attenuation owing to high altitude compared to the ground node. Therefore, a URN can provide superior performance in terms of radio coverage and link quality.

**Figure 1:**
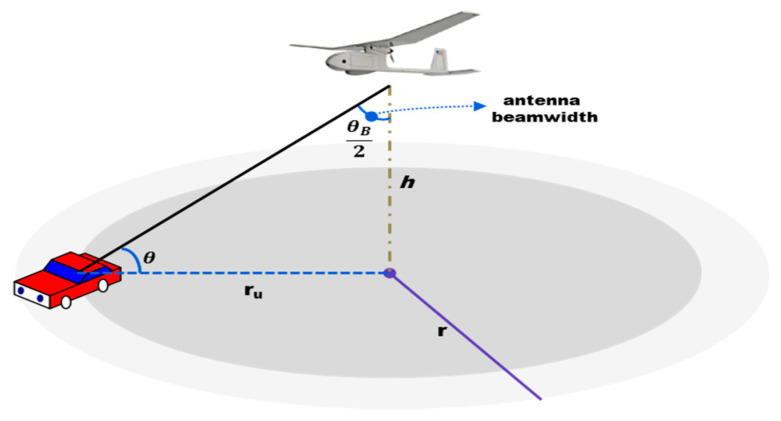
UAV relay coverage model.

Consider a circular geographical area of radius *r* as depicted in Figure 1 in which a UAV is deployed to provide wireless coverage for ground users located within the area. For air-to-ground channel modeling, a common approach is to consider the LoS and NLOS links between the UAV and the ground users separately [18]. The coverage probability ($P_{cov}$ [19]) for the ground node, located at a distance $r\leq r_{u}=htan\left(\frac{\theta_{B}}{2}\right)$ from the projection given $UAV_{j}$ in the area, is provided by Equation (2):

(2) $$P_{cov}=P_{LoS,j}\left(\frac{P_{min}+L_{dB}-P_{t}-G_{3dB}+\mu_{LoS}}{\sigma_{LoS}}\right)+\;P_{NLoS,j}(\frac{P_{min}+L_{dB}-P_{t}-G_{3dB}+\mu_{NLoS})}{\sigma_{NLoS}},$$

where $P_{min}=10\log\left(\beta N\right)$ is the minimum received power requirement for a successful detection, N is the noise power, and β is the signal-to-noise ratio (SNR) threshold. In addition, $L_{dB}$ is the path loss, and $G_{3dB}$ is the antenna gain ($G_{3dB}\approx 29000/\theta_{B}^{2}).$

Because 802.11p is expected to be widely used in industrial areas, and is the most suitable for VANET [20,21,22,23], we adopted the IEEE 802.11p MAC protocol for both inter-GVN communication and UAV-to-GVN communication.

We classified the following three types according to the characteristics of the data services based on the packet priority for an efficient operation of a URN in a congested network environment.

(1) Urgent service message (USM): Highest priority services that need to be urgently sent.

(2) Real time service (RTS): Medium-priority services with delay constraints but little packet loss.

(3) Connection oriented protocol (COP): Lowest priority services with less sensitivity to delay and loss.

In terms of network services, it is extremely important to select a routing path by considering traffic characteristics. From the user’s perspective, the effects experienced by a packet loss or delay are extremely different depending on the traffic characteristics. For example, there is a considerable difference between a streaming service that requires real-time and delay-insensitive TCP services.

## 4. Proposed Q-LBR Design

**Figure 2:**
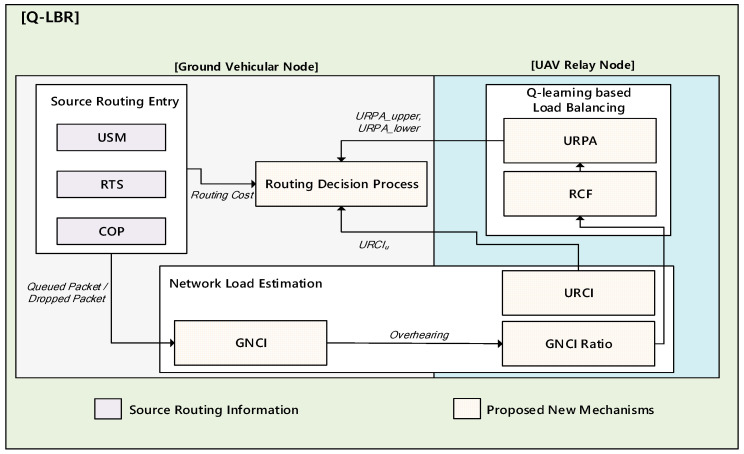
Q-learning based load balancing (Q-LBR) framework.

In this section, we describe the Q-LBR design in detail. Q-LBR is designed to maximize the network utilization of a URN through load balancing. Q-LBR introduces new mechanisms in UAV-assisted VANET, as described in Figure 2.

**Figure 3:**
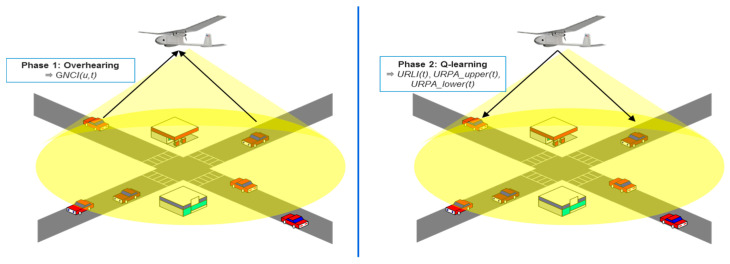
Two phases of Q-LBR.

The Q-LBR protocol consists of two phases, as described in Figure 3. During the first phase, a URN collects a ground network congestion identifier (GNCI) to the GN messages through broadcast and unicast overhearing to determine the congestion level of the ground network. Through this phase, the URN can recognize the congestion level of the ground network based on the collected GNCI and UAV relay congestion identifier (URCI) information. During the second phase, the URN disseminates URPA information corresponding to the action of the Q-learning. Specifically, the URN substitutes the GNCI and URCI into the Q-learning states and feeds the appropriate reward value back based on an RCF calculation. Finally, the result of the RCF determines the URPA value, which is divided into upper and lower values, and shares it with a Hello message.

### 4.1. Path Discovery and Maintenance

The path discovery of Q-LBR is performed by route request (RREQ) flooding, and the basic routing search method is similar to the source-based multipath routing protocol adopted in the existing VANET. The destination node responds to the RREQ, including the optimal and suboptimal paths, using a route reply (RREP) message. This increases survivability of the VANET routing through the use of suboptimal paths when the optimal path is disconnected. In the path-discovery process, the URN can receive multiple RREQs for the same destination from many GVNs, and thus the number of URN responses is limited. Through the Q-LBR path discovery process, the source node can acquire route information to the destination node, including a URN. Q-LBR periodically transmits a probe packet for routing updates to maintain the optimal and suboptimal paths. If all paths are disconnected, the intermediate node sends a route error (RERR) message to the source node.

### 4.2. Network Load Estimation

#### 4.2.1. Ground Network Congestion Identifier

It is extremely important to determine how a URN identifies ground network congestion according to the traffic load. In brief, each GVN estimates the GNCI from itself by using the queue load. This bitwise information is delivered to the URN using overhearing or broadcast messages. Then, URN computes the ratio of GNCI ($GNCI_{ratio}$) in its time interval by using the number of $GNCI_{i}$ instances with a value of ‘1’ from GVN *i*.

For a more detailed explanation, $q_{ground_{i}}\left(t\right)$, given by Equation (3), which indicates the queue load of each GVN, is calculated as the ratio of the maximum queue length ($MQL_{i}$) to the average queue length ($AQL_{i}\left(t\right)$) corresponding to time *t* of GVN *i*.

(3) $$q_{ground_{i}}\left(t\right)=\frac{AQL_{i}\left(t\right)}{MQL_{i}}$$

Based on the result of $q_{ground_{i}}\left(t\right)$, each GVN calculates the weighted moving average $Q_{ground_{i,k}}\left(t\right)$, given by Equation (4), in the window size *k* from GVN *i*.

(4) $$Q_{ground_{i,k}}\left(t\right)=\frac{\sum_{k=0}^{n}w_{k}q_{ground_{i}}\left(t-k\right)}{\sum_{k=0}^{n}w_{k}}$$

Each ground node *i* determines whether the result of $Q_{ground_{i}}\left(t\right)$ exceeds the GVN load threshold $Q_{ground_{th}}$, given by Equation (5), and marks the value of $GNCI_{i}\left(t\right)$ with a ‘1’ or ‘0’ in the packet header.

(5) $$GNCI_{i}\left(t\right)=\;\{\begin{array}{c} 1\;if\;Q_{ground_{i}}\left(t\right)>Q_{ground_{th}}\\ \;0\;otherwise\; \end{array}$$

The URN receives $GNCI_{i}\left(t\right)$ of each ground node through an overhearing or broadcast messages and then calculates $GNCI_{ratio}\left(t\right)$, given by Equation (6), which is the ratio of the congested GVN to the total number of GVNs, *N*.

(6) $$GNCI_{ratio}\left(t\right)=\frac{\sum_{i=0}^{N}GNCI_{i}\left(t\right)}{N\left(t\right)}$$

#### 4.2.2. UAV Relay Congestion Identification

The URCI, given by Equation (7), is calculated through the URN’s own queue load from the UAV relay node *u*.

(7) $$URCI_{u}\left(t\right)=\frac{AQL_{u}\left(t\right)}{MQL_{u}}$$

With $URCI_{u}$, however, it can be recognized that the closer $AQL_{u}\left(t\right)$ is to $MQL_{u},$ and when considering the load balancing aspect, the greater the throughput within the maximum range the UAV can accommodate.

### 4.3. Q-Learning-Based Load Balancing

#### 4.3.1. Q-Learning Design for UAV-Assisted Network

**Figure 4:**
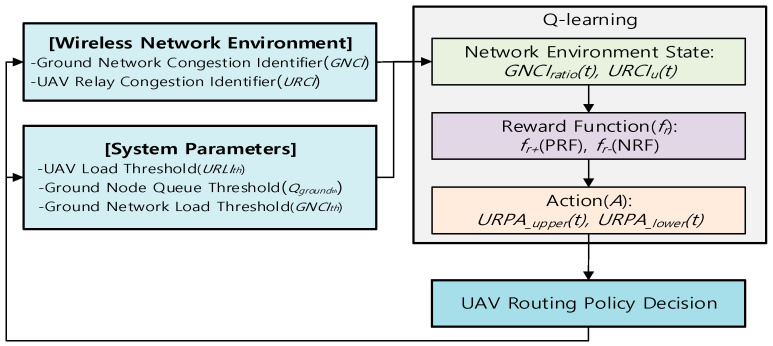
Q-learning design for Q-LBR.

Q-learning is a model-free reinforcement learning algorithm that finds an estimate of the optimal action-value function. It is able to compare the expected reward of the available actions for a given state without requiring a specific model of the network environment. Q-learning finds an optimal policy, in the sense that the expected value of the total reward return over all successive iterations is the maximum achievable. Figure 4 shows the Q-learning mechanism of the proposed method. 

**Figure 5:**
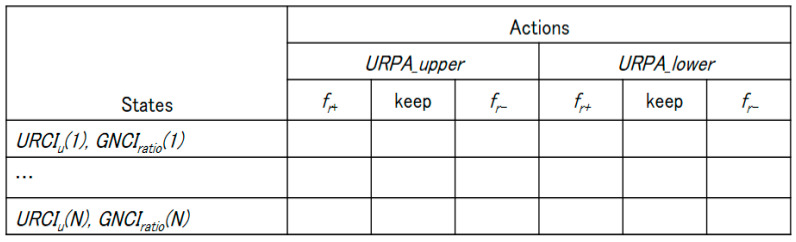
Q-table structure for Q-LBR.

An URN is an agent of Q-learning, and its action is a selection of URPA for the UAV routing policy decision. In Q-LBR, URN’s experience consists of a sequence of episodes. In the *N*_th_ episode, when URN finds a $URPA_{\_upper}$ and $URPA_{\_lower}$ that satisfies $URCI_{th}$ and $GNCI_{th}$, learning is terminated. If a network change occurs, and the $URCI_{th}$ and $GNCI_{th}$ are not satisfied, the learning process is repeated. Specifically, according to Figure 5 and Algorithm 1, the URN recognizes the wireless network environment through the $GNCI_{ratio}$ and $URCI_{u}$, then the URN learns in the network based on Q-learning and provides an appropriate reward *f_r_* according to $GNCI_{ratio}$ and $URCI_{u}$. The reward function *f_r_* selects *f_r+_*(PRF) in $URCI_{u}\left(t\right)\leq URCI_{th}$ situation and selects *f_r-_*(NRF) otherwise.

To recognize the state of the ground network, the URN listens to $GNCI_{i}$ transmitted from GVN *i* using an overhearing or broadcast messages. At time t, the URN can calculate $GNCI_{ratio}$ from the total number of N nodes. At the same time, the URN can calculate $URCI_{u}$ from its own queue load. The learning goal of Q-LBR is to find an optimal URPA that is as close as possible to $URCI_{th}$, which indicates the allowable load of the URN and satisfies an appropriate level of ground network load $GNCI_{th}$. If the URN finds the optimal URPA, the URN maintains its current state until it changes into a new network state. If not, the URN updates the Q-table according to the Q-learning procedure such that the reward value by the URPA actions can be maximized. Finally, the results of *URPA__upper_* and *URPA__lower_* corresponding to the action of the Q-learning are distributed to the GVNs. Through a repetitive execution of this process, the URN can find the optimal policy for the URPA suitable for the network environment.
**Algorithm 1: Q-learning based Load Balancing**1:URN ← UAV relay node;2:GVN ← Ground vehicular node;3:*GNCI_i_* ← Ground node congestion identifier from node i;4:*GNCI_ratio_* ← Ratio of congested GVNs;5:*URCI_u_* ← UAV relay congestion identifier from URN;6:*URCI_th_* ← Threshold of URCI;7:*URPA__upper_* ← Upper boundary value of UAV routing policy area;8:*URPA__lower_* ← Lower boundary value of UAV routing policy area;9:$f_{r}$ ← Reward function10:
11:**for**t → 1, n **do**12: **for**
i → 1, *N*
**do**13:   URN listens to $GNCI_{i}$ using overhearing or broadcast messages from node *i*14:   URN calculates $GNCI_{ratio}$ at time *t* received from total number of *N*15:   URN calculates $URCI_{u}$ at time t from its own queue load16:
17:   **if** ($GNCI_{ratio}<GNCI_{th}$ && $URCI_{u}≅URCI_{th}$) **then**18:    URN maintains its current state19:   
**Else**
20:    URN calculates the reward $f_{r}$(*t*-1) for the previous action *a*(*t*-1) at state s(*t*-1)21:    URN updates the Q-value of (*s*(*t*-1), *a*(*t*-1)) in Q-table22:    URN determines the current state *s*(*t*) based on the $GNCI_{ratio}$ and $URCI_{u}$
23:    URN selects the optimal action a(*t*) for the next *t*+1 time period24:
   **end if**
25:
26:   URN distributes $URCI_{u}$ and URPA__upper_ and URPA__lower_ to GVN27:
 **end for**
28:**end for**




#### 4.3.2. UAV Routing Policy Area

In a rapidly changing network environment, it is important to narrow and simplify the scope of the problem to be solved in order to design an optimal policy for an effective URN routing through the RL. If a learning algorithm is designed, including ground network routing, the problem to be solved becomes more complicated and the reward through the RL becomes difficult to effectively reflect. Therefore, Q-LBR defines URPA corresponding to two knobs (*URPA__upper_* & *URPA__lower_*) when considering the priority of traffic and the existence of a route independently from the ground network routing.

**Figure 6:**
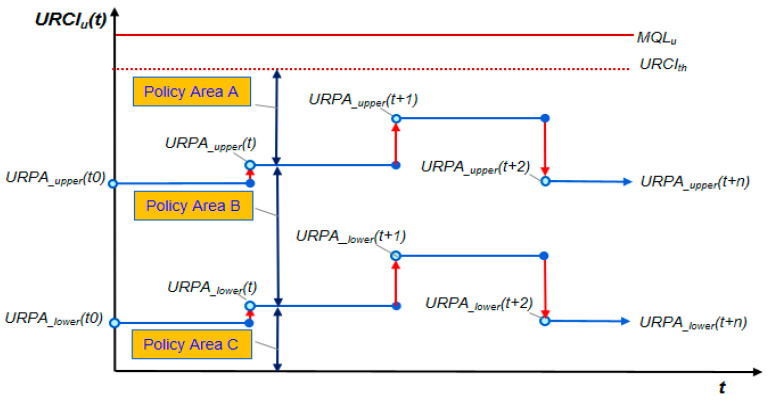
UAV routing policy area (URPA) example.

URPA is a parameter for applying the URN routing policy, and is defined in the following three policy areas to determine whether or not to be the route of an air node relay when a URN is present on the routing path of the GN. URPA sets the boundary for the policy area based on the parameters of *URPA__upper_* and *URPA__lower_* (*URPA__upper_ > URPA__lower_*), as shown in Figure 6, and dynamically changes with time *t* based on the action of the Q-learning.

- Policy Area A: Allow a UAV relay only when there is no ground path with a high-priority packet
- Policy Area B: Allow a UAV relay only when there is no ground path without considering the packet priority.
- Policy Area C: Allow a UAV relay without considering the packet priority or existence of the ground path (allow all traffic)

#### 4.3.3. Reward Control Function Design for Rapid Convergence

Reinforcement learning is a problem faced by an agent who must learn behavior through trial-and-error in a dynamic environment. However, the learning method can cause a convergence speed problem in terms of the time required to find the optimal state. In particular, the network environment is changed by various variables over time, and thus a method allowing the reinforcement learning system to respond quickly is required. Previous studies in which Q-learning was applied were generally proposed to control the learning rate through the value of *α*. However, if *α* is too large, it is difficult to converge to the optimal value function and, if it is too small, it takes too long to learn. This shows that there is a limitation in coping with rapid changes in the network with the existing method through the reflection ratio of the learned results. 

Q-LBR proposes using an RCF to determine the reward according to $URCI_{u}\left(t\right)$ and $GNCI_{ratio}\left(t\right)$ for the purpose of improving the convergence speed of the reward function. The RCF of Q-LBR dynamically determines the reward value according to the load-state of the URN and the ground network congestion with the rapidly changing network environment. Specifically, if the queue load of the URN is sufficient, a large positive reward value is given to increase the utilization of the URN. By contrast, under high congestion, a large negative value is given to reduce the URN and ground network congestion.

The reward function ($f_{r}\left(s_{t},\;a_{t}\right)$), given by Equation (8), is as follows:

(8) $$f_{r}\left(s_{t},\;a_{t}\right)=\{\begin{array}{cc} f_{r+}\left(s_{t},\;a_{t}\right) & if\;URCI_{u}\left(t\right)\leq URCI_{th}\\ f_{r-}\left(s_{t},\;a_{t}\right) & else \end{array}$$

The positive reward function (PRF), given by Equation (9), for action *a* is expressed as follows:

(9) $$f_{r+}\left(s_{t},\;a_{t}\right)=-1/ln(k*\left(1-\lambda \left(t\right)\right),$$

where λ(t), given by Equation (10), is a function (λ(t)∈(0,1]) for determining the reward values according to $URCI_{u}\left(t\right)$ and $GNCI_{ratio}\left(t\right)$ (where $URCI_{u}\left(t\right)\leq URCI_{th},GNCI_{ratio}\leq GNCI_{th}$). Here, *k* is the scale parameter (k > 0). When λ(t) is high, the reward value is significantly increased. When the value of λ(t) is low, it gradually increases.

(10) $$\lambda \left(t\right)=w_{1}*\left(\frac{URCI_{u}\left(t\right)}{URCI_{th}}\right)+w_{2}*\left(\frac{GNCI_{ratio}\left(t\right)}{GNCI_{th}}\right)$$

The negative reward function (NRF) for action a is expressed as follows:

(11) $$f_{r-}\left(s_{t},\;a_{t}\right)=ln(k*\left(1/r_{max}-\lambda \left(t\right)\right),$$

where $r_{max}$ is the maximum reward value ($r_{max}>\lambda \left(t\right),\;r_{max}>0)$. The NRF is also controlled by λ(t) and the weight w of URLI(t) and GNCI(t). In contrast to the PRF, when λ(t) is high, the reward value is significantly decreased, and when the value of λ(t) is low, the reward value gradually decreases.

### 4.4. Routing Decision Process

According to Algorithm 2, the ground source node can receive *p* messages owing to multiple paths from the ground destination node. Through this message, routing metrics are calculated in $RREP_{p}$ packets for each routing path. If $RREP_{p}$ including an URN exists, and this path is less expensive than the ground path, the $URCI_{u}$ of the URN and the traffic priority (TP) of the packets check the URPA condition. If all the conditions are satisfied, the path including the URN can be selected as the optimal path. If unsatisfied, the next suboptimal ground path is selected.
**Algorithm 2: Routing Decision Process**1:S ← Ground source node;2:D ← Ground destination node;3:URN ← UAV relay node;4:RCU ← Routing cost including UAV path;5:RCG ← Routing cost with GVN only;6:URPA ← UAV routing policy area;7:*URCI_u_* ← UAV relay congestion identifier from URN;8:TP ← Traffic priority9:
10:**for***p* → 1, n **do**11: **if** S receives $RREP_{p}\left(D\right)$ packet **then**12:  Calculate routing cost using metric information collected in $RREP_{p}\left(D\right)$ packet13:  **if** ($RREP_{k}$ path contains URN || RCU < RCG) **then**14:    **if** ($URCI_{u}$ and TP satisfy URPA’s UAV relay conditions) **then**15:     Select the routing path that includes the URN as the optimal route16:
    **else**
17:     Select the suboptimal ground path18:
    **end if**
19:
  **Else**
20:   Select the optimal ground path21:
  **end if**
22:
 **end if**
23:**end for**





## 5. Simulation Results and Analysis

### 5.1. Simulation Environments

In this section, we evaluate the performance of the proposed protocol using the network simulator Riverbed Modeler version 18.7. We summarize the detailed information regarding our simulation parameters in Table 2. 

During the simulation, three types of packets are considered: USM, RTS, and COP packets. USM is a traffic type corresponding to the emergency data and control message of a critical service, and is set to EF, the highest packet priority. The size of the USM packet is set to 256 bytes based on an exponential distribution, and the packet interval is set to 10 requests per second (r/s).

Since the traffic size and request rate follow the exponential distribution $f_{X}$ with parameter $\lambda_{s}$ as follows:

(12) $$f_{X}\left(x\right)=\lambda_{s}e^{-\lambda x}$$

RTS is a traffic type corresponding to a service requiring a certain amount of real-time data using a codec such as a video stream. The priority of the RTS packet is set to AF21, which is the middle priority of the packet. The size of the RTS packet is set to 1500 bytes, and the packet interval is set to 10 r/s. COP is a traffic type corresponding to TCP data, such as FTP, and is set to CS0, the lowest packet priority. The size of the COP packet is set to 256 bytes based on an exponential distribution the same as USM, and the packet interval is set to 10 r/s. To support the QoS requirements for different services, the IEEE 802.11p EDCA mechanism defines four access categories (AC0–AC3) for each channel. We defined AC0 through AC2 for mapping to USM, RTS, and COP services, respectively. The arbitration interframe space (AIFS) is determined according to the mapping relationship for each service. AIFS indicates the idle channel time that must be endured for a transmission opportunity.

**Figure 7:**
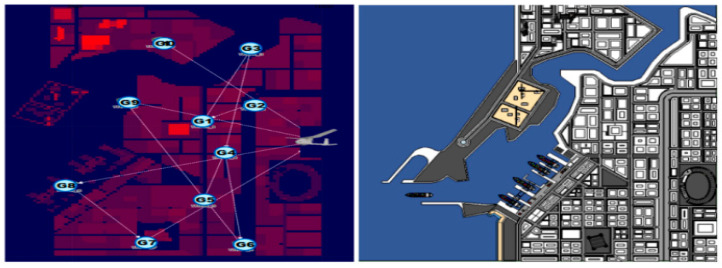
Basic network layout (10 GVNs and 1 URN) in the Riverbed Modeler.

The overall network layout in the Riverbed Modeler is shown in Figure 7. We applied the urban propagation model provided by the Riverbed Modeler when considering the network connectivity from the building attenuation effect. Initially, 11 radio nodes (10 GVNs and one URN) are deployed within a 1000 m × 1000 m region. Each GVN is randomly placed, and the random way point (RWP) model is applied as the mobility model.

Each GVN generates bidirectional USM, RTS, and COP packets, and each GVN establishes a pair with a random destination for three traffic pairs. The URN performs only the relay role and does not generate traffic except for the routing control message. We conducted the simulation 100 times with a 95% confidence interval.

### 5.2. Perforamnce Analysis

The key element of Q-LBR is URPA, which induces a load balancing between the URN and the ground network. GNs determine the routing according to the URN load and ground network load based on the URPA. Therefore, if the URN grants the maximum allowable traffic through the proper URPA, a positive effect on the overall network performance can be expected because the URN path can provide a higher quality clear-LoS link than the ground path.

**Figure 8:**
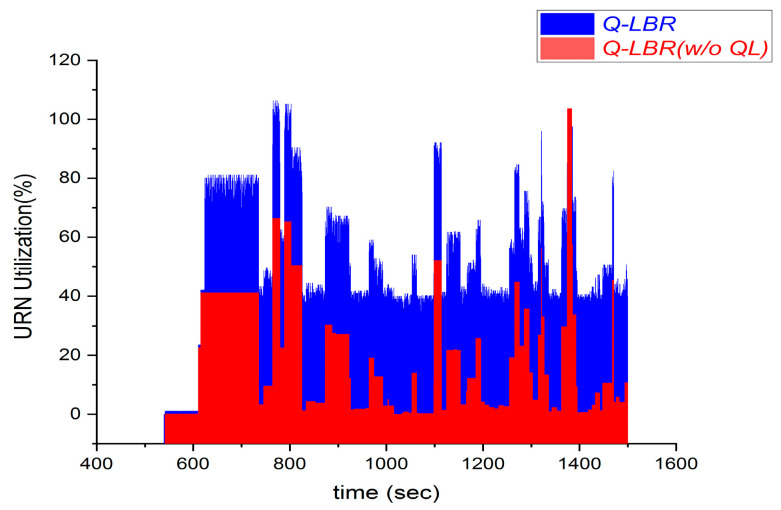
Q-LBR versus Q-LBR without Q-Learning for URN utilization.

Figure 8 shows the results of a comparative experiment when setting the URPA as a fixed value without a learning process and assigning a dynamic value through Q-learning from the perspective of the URN utilization ($Q_{ground_{th}}$= 70, $URCI_{th}$= 80, $GNCI_{th}$= 50, $w_{1}$ = 0.7, and $w_{2}$= 0.3). URN utilization is a performance index that indicates the average queue length compared to the maximum queue length of the UAV per unit time, and is the same as $URCI_{u}$, which indicates the queue load of the URN. This metric shows the degree of URN utilization in the network. A lower URN utilization means that $URCI_{u}\left(t\right)$ is low because the load on the URN is idle. By contrast, in the case of the same traffic condition, a higher URN utilization means the UAV load is close to the maximum allowable queue length, and thus the URN is busy. However, if $MQL_{u}$ is exceeded, it means that a queue drop occurs, and thus it is necessary to set the appropriate $URCI_{th}$.

In the case of Q-LBR (w/o QL), a fixed URPA policy is applied, and thus there is no coordination according to the ground network load and URN load conditions. Therefore, the overall URN utilization is relatively low (1%–40%). In the case of Q-LBR with Q-learning, the result shows that the URN utilization by dynamic URPA is improved by Q-learning. Therefore, the overall URN utilization is relatively high (40%–80%). If $URCI_{th}$ and $GNCI_{th}$ are increased, a higher URN utilization can be expected in Q-LBR with Q-learning. However, as the URN utilization increases, the possibility of a packet loss owing to an overload increases proportionally, and thus it is necessary to set an appropriate level (70%–80%). As a result, this experimental result shows that Q-LBR with Q-learning has a significant effect on the dynamic URPA

**Figure 9:**
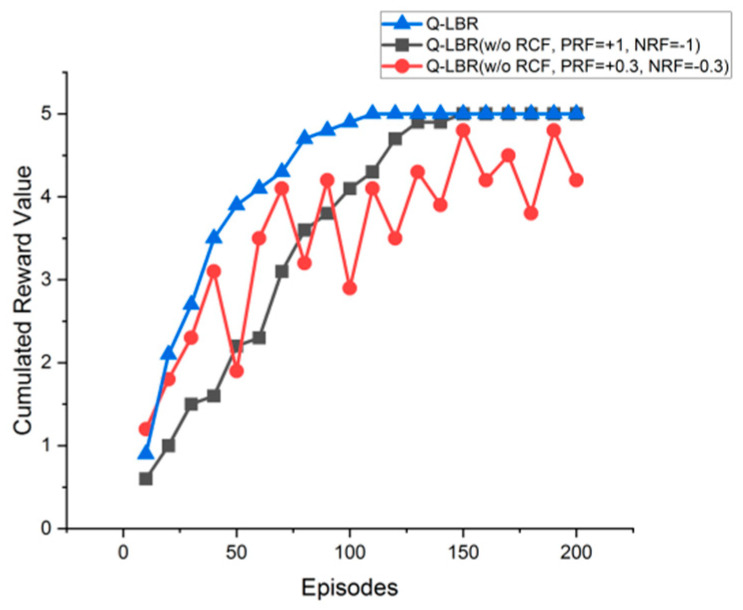
Q-LBR versus Q-LBR without Reward Control Function (RCF) for Cumulated Reward Value.

Figure 9 shows the results of the comparative experiment according to the RCF of Q-LBR in the same environment as the above experiment. The purpose of the experiment was to find out how RCF affects the convergence speed through cumulated reward value (CRV). As a result, it was confirmed that there was a difference in the number of episodes required to reach the maximum reward value ($r_{max}\;$= 5) depending on whether or not RCF or the reward value. Q-LBR (w/o RCF, PRF=+1, NRF=−1) approached $r_{max}$ most quickly in the first 10 to 70 episodes, but the results were not converged even after 200 episodes. Q-LBR (w/o RCF, PRF = +0.3, NRF = −0.3) showed convergence after about 160 episodes. This result shows that the probability r_max of is high when the fluctuation of the reward value is small, but the probability of increasing the number of required episodes is high. On the other hand, since Q-LBR (with RCF) adjusted the reward value adaptively in consideration of the ground network load and URN load, it showed a rapid increase in the beginning and converged in a gentle curve. Finally, it converged to 110 episodes, which decreased by about 32% compared to Q-LBR (w/o RCF, PRF = +0.3, NRF = −0.3).

**Figure 10:**
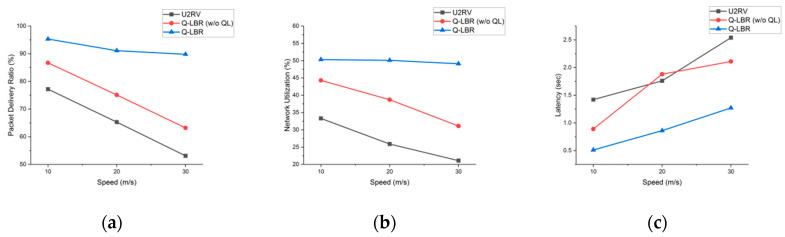
Performance comparison for ground node speed: (**a**) total PDR, (**b**) total network utilization, and (**c**) total latency.

From Figure 10 and Table 1, we can see that as the node speed increases the packet loss rate of the Q-LBR is lower than that of U2RV. Q-LBR also performs better in terms of network utilization and latency. As the speed of the GVN increases, the probability of the topology changing increases and retransmission by routing control messages and route disconnection increases. U2RV is a multi-criteria routing protocol based on segment density and distance. This protocol only considers the possibility of increasing the traffic through the segment density and does not consider the actual user traffic that may occur in each GVN. In particular, it can be seen that an increase in retransmissions due to a topology change under the same URN coverage may degrade the total network performance. 

Q-LBR (w/o QL) is the result of setting a fixed URPA value (*URPA__upper_* = 60, *URPA__lower_* = 10), except for the Q-learning process. Compared to U2RV, although there is an improvement in performance owing to traffic distribution, a problem occurs in that it is not possible to increase the utilization of the URN by adapting to changes in the network environment. The resulting latency is compared with that of U2RV (20 m/s) in Figure 10c. Based on this result, it can be seen that the fixed URPA may not be properly adapted to the network environment under certain situations.

By contrast, Q-LBR shows that it can cope with topology changes caused by network mobility through Q-learning. Q-LBR enables the URPA value to be adaptive to the network situation based on the learning process through RCF. As a result, the changing trend of the graph as the speed increases shows a rather gentle curve compared to the other results. In Table 3, Q-LBR shows a lower COP performance than that of U2RV. This is because COP packets are dropped under congestion or routed only through the ground path by the dynamic URPA. From a system perspective, because COP is a service that is less sensitive to delay and loss, it is reasonable to prioritize USM and RTS. Based on a moving speed of 30 m/s and total traffic flows, Q-LBR shows a PDR of approximately 89.8%, network utilization of 49.1% and latency of 1.27 s.

**Figure 11:**
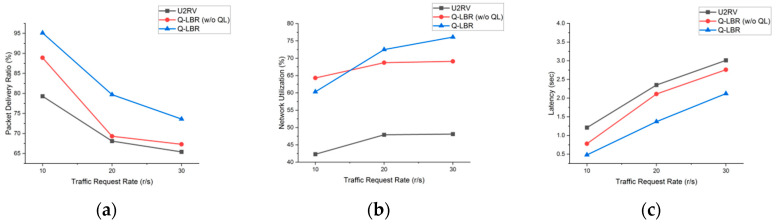
Performance comparison for traffic request rate: (**a**) total PDR, (**b**) total network utilization and (**c**) total latency.

Figure 11 and Table 4 show the performance results in terms of the traffic request rate (requests/s), which were similar to those obtained in a previous simulation. However, in the case of a large number of traffic requests exceeding the network capacity, the load balancing efficiency is reduced owing to the multihop resource occupancy of low-priority traffic. This result shows that the dropping of packets in the first hop of the bottleneck link through the URN is more advantageous than dropping through a multihop ground relay. This problem can be solved using the QoS technique (e.g., shaping or policing) to limit the amount of traffic output transmitted with a low priority. Based on the traffic request rate of 30 r/s and the total traffic flows, Q-LBR shows a PDR of approximately 73.6%, a network utilization of 76.1% and a latency of 2.12 s. As the amount of traffic increases, the overall performance is lowered compared to the previous experiment, but still shows a stable performance based on dynamic load balancing. 

## 6. Discussions

In this chapter, we discuss the feasibility in a real-world scenario of this study. Q-learning faces a problem of memory and high computation requirements if the combination of states and actions are too large. In this paper, network simulation was performed based on 10 GVNs and 1 URN. Computational operations related to Q-learning were performed entirely by URN and there was no problem in running the simulation. However, if the size of the network increases and the number of Q-learning actions increases, the size of the Q table becomes extremely large. In this case it may not be possible to apply the Q-learning algorithm because of the URN’s computational power. In particular, the communication hardware mounted on URN is an embedded system and there are limitations on memory and power. As a solution to this, deep reinforcement learning (DRL), which combines deep learning and reinforcement learning, is considered to be an effective alternative. For example, multistep learning- Deep Q-learning Network (DQN) [24] proposed the concept of using multilayered compensation after a one-step bootstrap when calculating the target Q value. If Q-learning is performed in advance by using the reward information after an n-step bootstrap, it is expected that the amount of computation required for learning can be greatly reduced.

## 7. Conclusions

In this paper, we proposed a new UAV-assisted routing protocol, called the Q-LBR, that uses a Q-learning algorithm to handle UAV relay traffic. The proposed protocol uses an URPA mechanism when considering the traffic priority and the existence of a route independently from ground network routing. We also proposed an RCF for rapid learning feedback of the reward values in consideration of a dynamic network environment. Q-LBR adjusts the reward value according to the URN load and ground network congestion. Performance evaluation using the Riverbed Modeler showed that Q-LBR achieved a significantly better network throughput and latency compared to that of existing algorithms. As a continuation of this work we plan to continue research on implementation of actual equipment and additional algorithms linked to DRL.

## Figures and Tables

**Table 1:** Comparative study between routing protocols.

Features	LCAD ^1^	U2RV ^2^	UCLR ^3^	HRLB ^4^	Q-LBR ^5^
Multipath	No	Yes	Yes	Yes	Yes
UAV-assisted Relay	Yes	Yes	Yes	No	Yes
Traffic Characteristics	No	No	No	No	Yes
Load Balancing	No	No	Yes	Yes	Yes
Dynamic Load Control	No	No	No	No	Yes
Machine Learning	No	No	No	No	Yes
Type of network	UAV/MANET	UAV/VANET	UAV/MANET	VANET	UAV/VANET

^1^ LCAD: Load Carry and Deliver Routing. ^2^ U2RV: UAV-assisted Reactive Routing Protocol for VANET. ^3^ UCLR: UAV-aided Cross-Layer Routing. ^4^ HRLB: Hierarchical Routing Scheme with Load Balancing. ^5^ Q-LBR: Q-learning based Load Balancing Routing.

**Table 2:** Simulation Parameters.

Layers	Parameters	Settings
PHY	Data Rate	1 Mbps
	Propagation Loss Model	Urban Propagation Model
	Coverage Probability (Air-to-Ground)	$P_{cov}$ [13]
	Frequency Band	5.9 GHz
MAC	Protocol	802.11p
	Slot Time	13 μs
	SIFS	32 μs
	AIFNSN[AC0:USM/AC1:RTS/AC2:COP]	2, 3, 6
Network	Hello Interval	30 s
	Active Route Timeout	90 s
Application	USM Traffic (Size/Rate)	Exp. 256 bytes/Exp. 10 rps
	RTS Traffic (Size/Rate)	Con. 1500 bytes/Con. 10 rps
	COP Traffic (Size/Rate)	Exp. 256 bytes/Exp. 10 rps
Q-learning	Learning rate(α)	0.3
	Discount Factor(γ)	0.7
	$r_{max}$	5
UAV	Altitude Antenna	1000 m Omni-directional

**Table 3:** Simulation results for varying the speed of the nodes (all traffic = 10 r/s).

Protocol	Speed (m/s)	Traffic Type	Packet Delivery Ratio (%)	Network Utilization (%)	Latency (s)
U2RV	10	USM	78.3	33.6	1.3
		RTS	77.1		1.5
		COP	79.7		1.6
	20	USM	65.4	27.3	1.5
		RTS	69.1		1.8
		COP	67.3		1.9
	30	USM	66.7	24.3	2.1
		RTS	63.5		2.7
		COP	61.1		2.8
Q-LBR	10	USM	83.1	44.8	0.8
(w/o QL)		RTS	82.4		1.1
		COP	50.5		1.2
	20	USM	81.7	42.1	1.4
		RTS	78.5		1.9
		COP	52.1		2.1
	30	USM	72.1	28.6	1.9
		RTS	68.5		2.1
		COP	58.7		2.2
Q-LBR	10	USM	93.3	50.3	0.5
		RTS	91.1		0.8
		COP	67.5		1.8
	20	USM	92.6	50.1	0.6
		RTS	90.8		0.9
		COP	62.1		2.3
	30	USM	92.5	49.8	0.8.
		RTS	89.7		0.9
		COP	61.8		2.8

**Table 4:** Simulation results for varying the traffic request rate (speed = 0 s).

Protocol	Traffic (r/s)	Traffic Type	Packet Delivery Ratio (%)	Network Utilization (%)	Latency (s)
U2RV	10	USM	79.2	42.8	1.24
		RTS	76.3		1.31
		COP	78.1		1.35
	20	USM	68.5	47.7	2.11
		RTS	67.6		2.35
		COP	68.1		2.42
	30	USM	64.3	47.9	2.72
		RTS	66.5		2.77
		COP	66.8		2.83
Q-LBR	10	USM	88.9	64.9	0.81
(w/o QL)		RTS	86.5		0.92
		COP	75.4		0.98
	20	USM	76.3	67.4	1.98
		RTS	72.7		2.37
		COP	64.5		2.61
	30	USM	69.2	67.3	2.57
		RTS	65.2		2.81
		COP	60.7		2.99
Q-LBR	10	USM	96.7	60.8	0.44
		RTS	94.2		0.56
		COP	64.3		0.99
	20	USM	87.4	72.5	1.18
		RTS	83.5		1.22
		COP	62.2		2.76
	30	USM	77.5	75.9	1.39
		RTS	74.1		1.98
		COP	61.9		2.95
